# Vascular Leiomyoma Presenting as Anterior Knee Pain (Case Report)

**DOI:** 10.5334/jbr-btr.976

**Published:** 2016-03-15

**Authors:** Elçin Aydın, Gokcen Coban, Esra Zeynep Coşkunoğlu, Mehmet Tükenmez

**Affiliations:** 1 Başkent University, TR

**Keywords:** vascular leiomyoma, angioleiomyoma, subcutaneus tumours, MRI

## Abstract

Vascular leiomyomas or angioleiomyomas are rare benign solitary smooth muscle tumors that origin usually in the extremities. Most of these tumors are composed of venous vessels. Here in, we report a rare case of subcutaneous vascular leiomyoma of the right knee of a 38 year old woman who was presented with recurrent anterior right knee pain and soft tissue swelling. Clinical findings, magnetic resonance imaging and histopathologic findings of the tumor is discussed. Leiomyomas are not mostly considered in the differential diagnosis by radiologist due to its rarity. Typical imaging and clinical findings of a tumor is an important clue for an accurate and early diagnosis.

## Background

Angioleiomyomas or vascular leiomyomas are rare subcutaneus benign tumours arising from smooth muscle cells of arterial or venous walls [1]. They are most frequently seen in women and in the lower extremities [2,3]. The typical presentation is a painful subcutaneus mass. Magnetic resonance imaging (MRI) and clinical findings help with the diagnosis. Our aim is to present a rare case of vascular leiomyoma in the right knee of a 38-year-old woman with MRI, clinical, and pathological findings.

## Case Presentation

**Figure 1:**
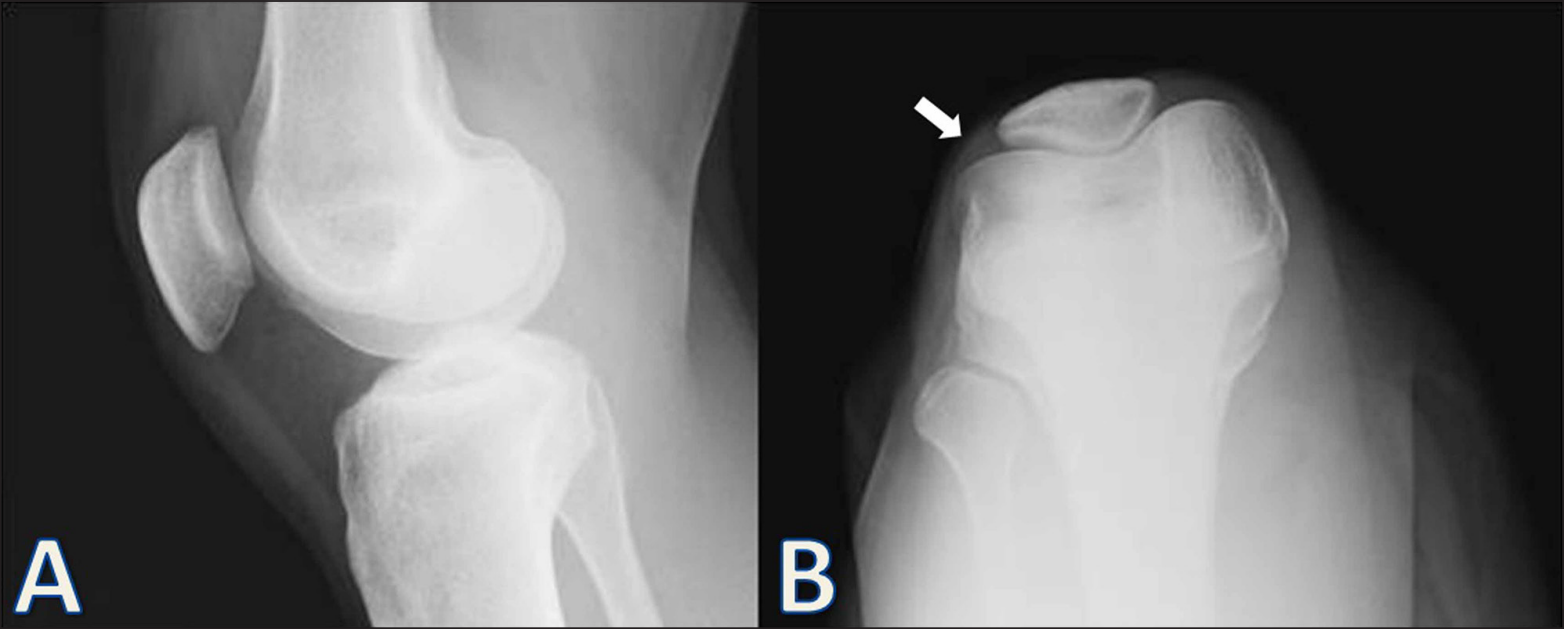
Lateral (A) and skyline (B) plain orthogonal radiographs of the right knee demostrate no bone abnormalities. The soft tissue mass (white arrow) is best seen on the skyline radiographic view.

**Figure 2:**
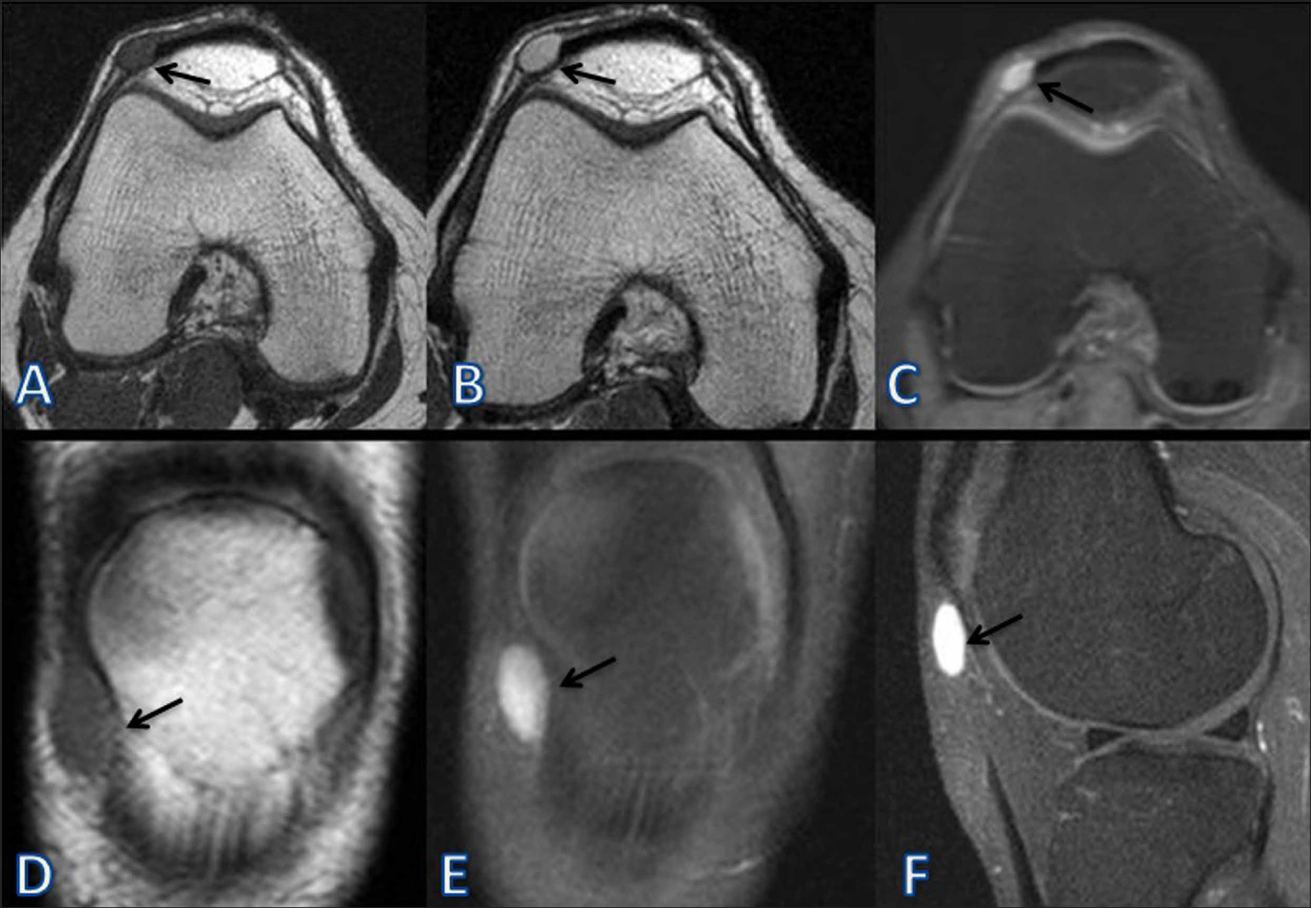
This is a preoperative MRI of the right knee. Axial (A) and coronal (D) T1-weighted pre-contrast images show well-circumscribed, hypointense soft tissue lesion adjacent to the lateral patellar retinaculum. The axial PD-weighted imaging with fat suppression (C) image shows a well-circumscribed, marked homogenous hyperintense subcutaneus lesion. Post-contrast axial (B), fat-supressed coronal (E), and sagital (F) T1-weighted images show prominent enhancement of the tumor and a sharp outline.

A 38-year-old woman presented with painful, mobile swelling in the right knee. There was no trauma history, and the onset of pain was sporadic and had reduced her mobility. On physical examination, there was a mobile painful swelling on the lateral aspect of her right knee. The laboratory findings were normal. Plain orthogonal radiographs including lateral and skyline views of the knee demostrated no bone abnormalities (Figure 1A–B). But there was a soft tissue swelling on the lateral aspect of the patella (Figure 1B). MRI examination with and without contrast was performed. An MRI of the right knee revealed a subcutenous, ovoid, well-circumscribed, homogenous, capsulated soft tissue mass adjacent to the lateral patellar retinaculum (Figure 2A–F). The tumor was hyperintense on proton density (PD) images and hypointense on T1-weighted images according to muscles. On post-contrast T1-weighted (Figure 1B) and PD-weighted (C) images there was a sharp thin hypointense rim surrounding the lesion. The tumor showed marked homogeneous gadolinium enhancement after contrast administration (Figure 1B, E–F). There was no joint effusion or soft tissue edema. The muscles and bones were normal.

**Figure 3:**
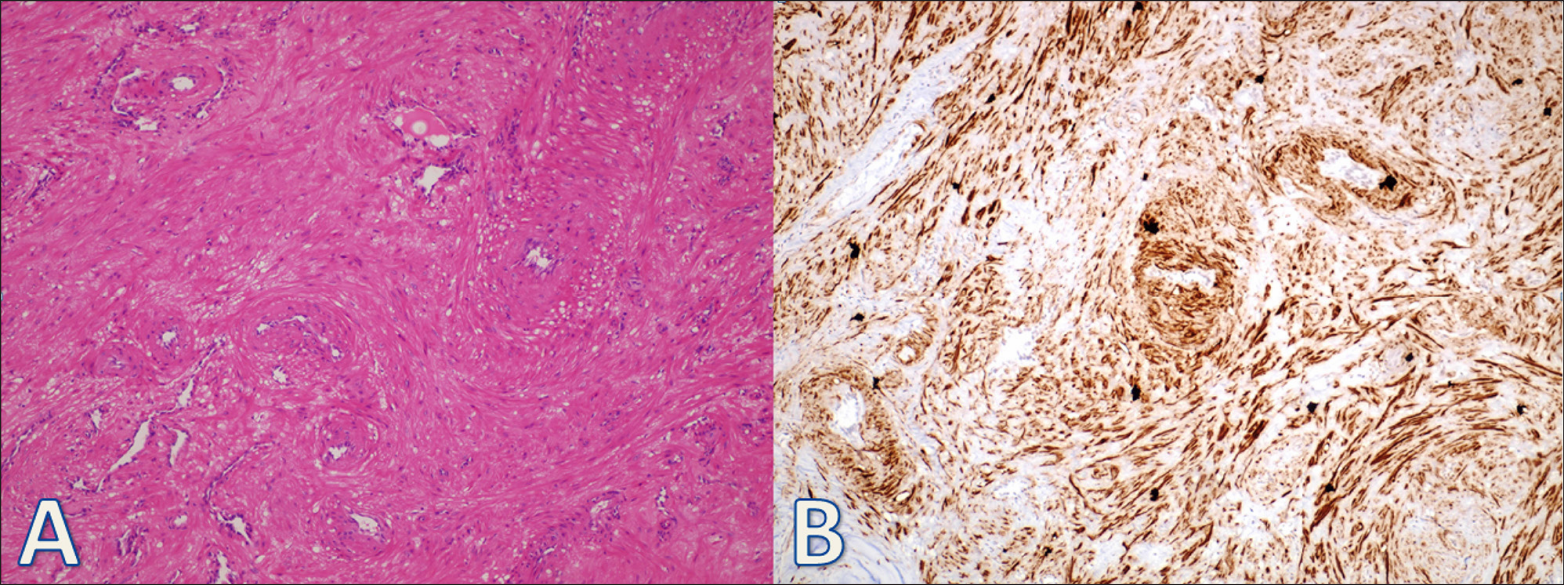
The tumor is composed of well-differentiated smooth muscle cells and blood vessels ((A), H&E; 200). Immunohistochemical staining for SMA shows positive reaction in smooth muscle bundles ((B)×200).

The patient underwent surgery and complete excision of the tumor was performed. Gross examination revealed a 10×10 mm firm, well-circumscribed, bean-shaped mass with a white-beige cut surface. Histological sections demonstrated a solid tumor composed of intersecting fascicles of mature smooth muscle cells surrounding vascular structures (Figure 3A). The smooth muscle cells showed no cellular atypia, and mitotic figures were rare. Immunohistochemical analysis showed diffuse positivity for smooth muscle actin (SMA) (Figure 3B). After the operation, her symptoms disappeared.

## Discussion

Vascular leiomyomas or angioleiomyomas are benign subcutaneus tumours that originate from the smooth muscles of blood vessels. They commonly influence the lower extremities, but they seldom affect the knee [1,2]. Females are more likely to be affected than males; vascular leiomyomas generally occur in the third or fourth decads [2,3]. Angioleiomyomas are generally seen in the deep layers of the dermis or in the subcutaneus tissue. Histologically, angioleiomyomas consist of smooth muscle bundles, vascular channels, and a thin fibrous capsule.

Morimoto defined three subtypes: solid or capillary, cavernous, and venous [3,4]. Solid is the most frequent, three times as common as in females and typically seen in the lower extremities. The cavernous subtype is more common in males than females and involves the head and upper extremities [4,5]. The most characteristic complaints are pain and tenderness. A painful subcutaneus mass in the lower extremities is the typical finding in the solid histological subtype. Differential diagnoses of a leiomyoma are glomus tumor, hemangiomas, angiolipoma, ganglion, schwannoma, giant cell tumor, neurilemoma, traumatic neuromas, and eccrine spiradenoma [4]. Small malignant lesions cannot be excluded by clinic or MRI alone.

Ultrasound remains the first-line imaging tool to confirm the presence of a mass lesion and its solid nature. MRI is the best choice for imaging because it can better delineate the lesion and define its relationship to the adjacent structures. Hwang et al. defined that hyperintens areas on T2-weighted MR images show the smooth muscle and numerous vessels in vascular leiomyomas [6]. The fibrous capsule defined as a hypointense rim on T2-weighted MRI, with contrast it has marked enhancement that shows its vascular origin [4,5,6,7].

In conclusion, when a painful subcutaneus swelling in lower extremities is seen, vascular leiomyoma must be kept in mind for the differential diagnosis. MRI is the best choice for imaging that can delineate the lesion and define its relationship to the adjacent structures, allowing better pre-excisional planning.

Leiomyomas are not often considered in the differential diagnosis by radiologists due to their rarity. They are usually well-circumscribed, sharply outlined, homogenous, and show prominent enhancement. Typical imaging and clinical findings of a tumor are important clues for an accurate and early diagnosis.

## Competing Interests

The authors declare that they have no competing interests.
